# Supplementary material for: Association Analysis of Salt Tolerance in Asiatic cotton (Gossypium arboretum) with SNP Markers
Source: Int J Mol Sci. 2019 May 1;20(9):2168. doi: 10.3390/ijms20092168 (PMC6540053; doi:10.3390/ijms20092168)
Supplement: Supplementary file 1 [file ijms-20-02168-s001.zip › Supplementary Figures S1-S3.docx]

**Supplementary Materials**


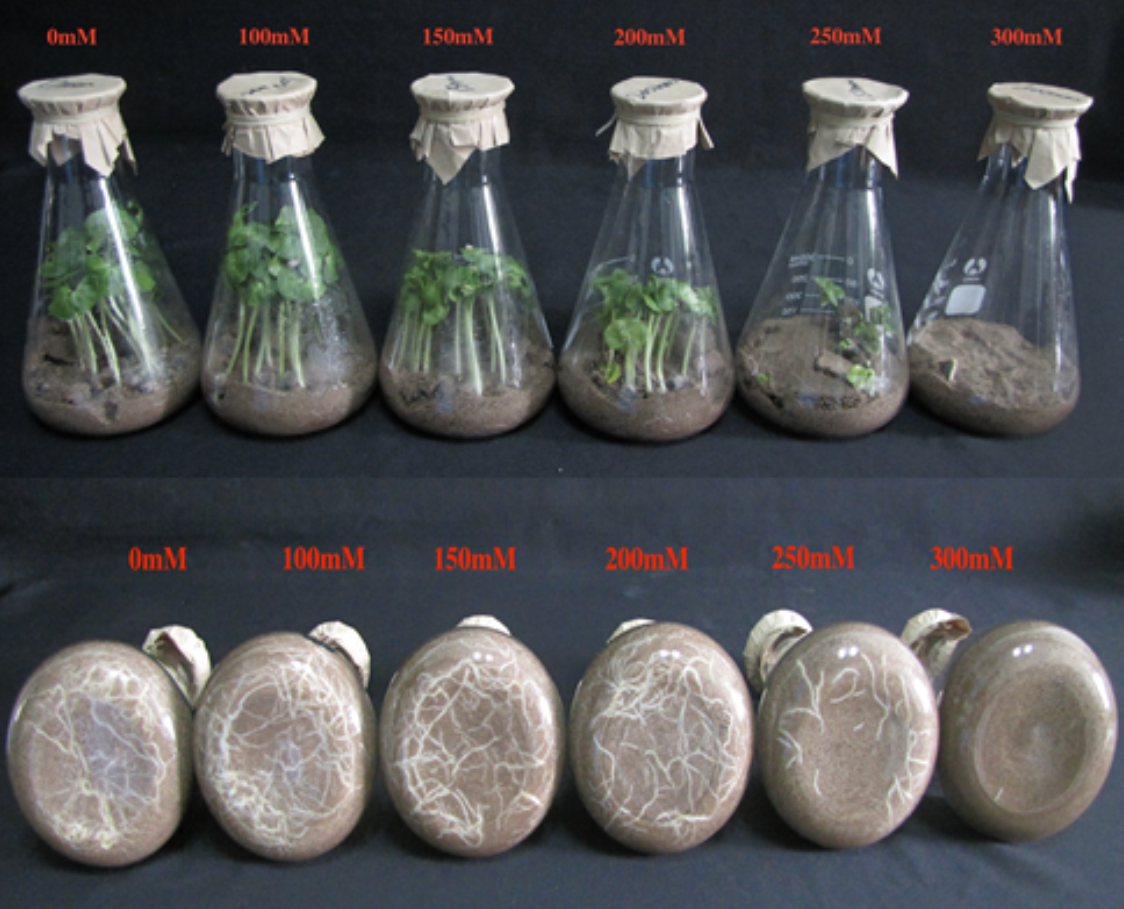


**Supplementary Figure S1**. The germination of Sichuansuiningzhongzianzuangzi under different NaCl concentrations (0, 50, 100, 150, 200, 250, and 300 mM) after 7 days of seed growth.


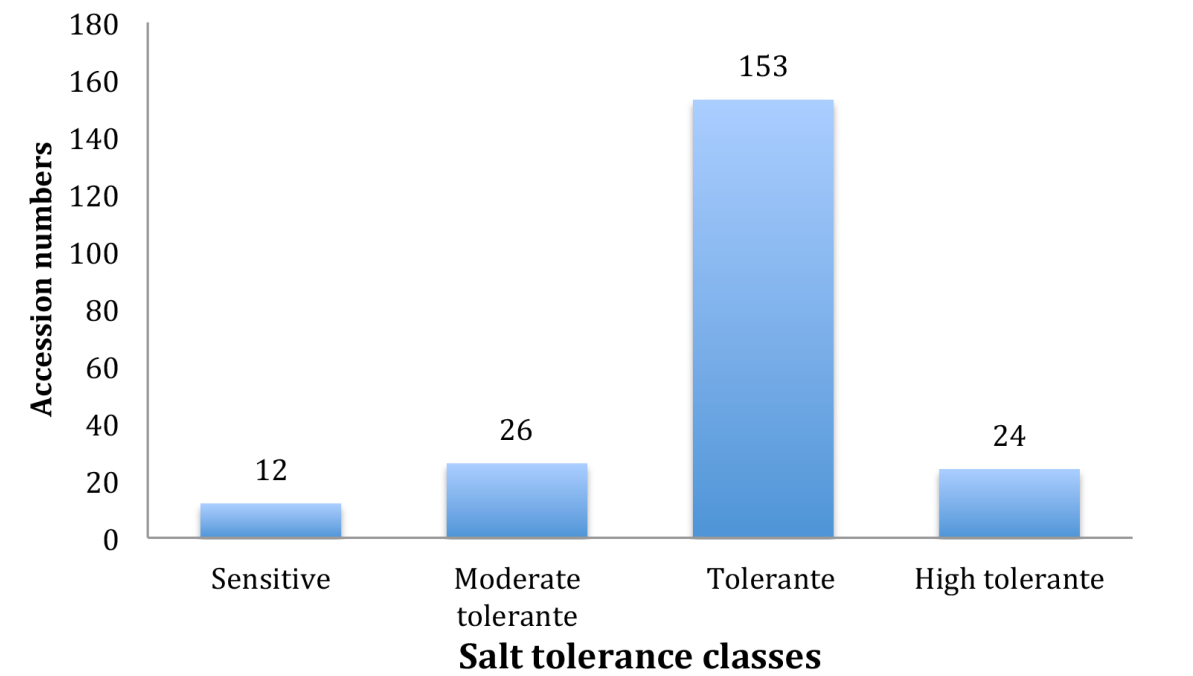


**Supplementary Figure S2.** Salt tolerance classifications of 215 *G. arboretum* accessions based on the comprehensive index of salt tolerance (CIST).


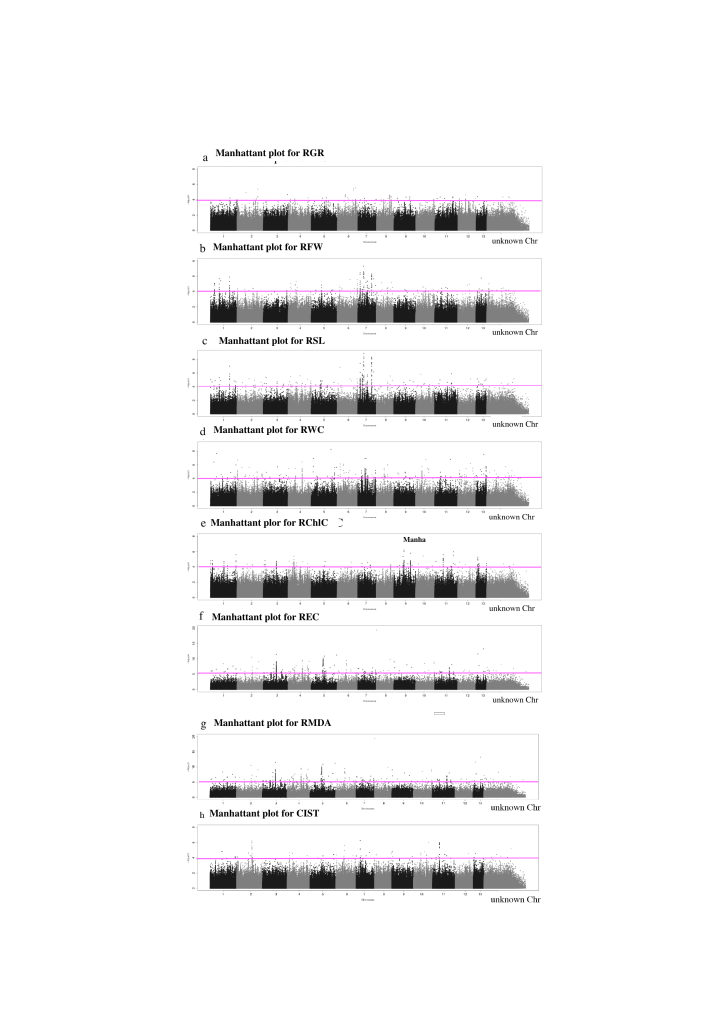


**Supplementary Figure S3**. Manhattant plots for seven salt-tolerance-related traits and the comprehensive index of salt tolerance (CIST) in 215 of *G. arboreum* accessions.
